# Intense Sweeteners, Taste Receptors and the Gut Microbiome: A Metabolic Health Perspective

**DOI:** 10.3390/ijerph17114094

**Published:** 2020-06-08

**Authors:** Alexandria Turner, Martin Veysey, Simon Keely, Christopher J. Scarlett, Mark Lucock, Emma L. Beckett

**Affiliations:** 1 School of Environmental and Life Sciences, University of Newcastle, Ourimbah 2258, Australia; alexandria.turner@uon.edu.au (A.T.); c.scarlett@newcastle.edu.au (C.J.S.); mark.lucock@newcastle.edu.au (M.L.); 2 School of Medicine and Public Health, University of Newcastle, Ourimbah 2258, Australia; martin.veysey@hyms.ac.uk; 3 Hull York Medical School, University of Hull, Hull HU6 7RX, UK; 4 School of Biomedical Sciences and Pharmacy, University of Newcastle, Callaghan 2308, Australia; simon.keely@newcastle.edu.au; 5 Hunter Medical Research Institute, New Lambton Heights 2305, Australia

**Keywords:** sweetener, non-nutritive sweetener, taste receptor, gut microbiome, obesity, metabolism, gut hormone

## Abstract

Intense sweeteners (IS) are often marketed as a healthier alternative to sugars, with the potential to aid in combating the worldwide rise of diabetes and obesity. However, their use has been counterintuitively associated with impaired glucose homeostasis, weight gain and altered gut microbiota. The nature of these associations, and the mechanisms responsible, are yet to be fully elucidated. Differences in their interaction with taste receptors may be a potential explanatory factor. Like sugars, IS stimulate sweet taste receptors, but due to their diverse structures, some are also able to stimulate bitter taste receptors. These receptors are expressed in the oral cavity and extra-orally, including throughout the gastrointestinal tract. They are involved in the modulation of appetite, glucose homeostasis and gut motility. Therefore, taste genotypes resulting in functional receptor changes and altered receptor expression levels may be associated with metabolic conditions. IS and taste receptors may both interact with the gastrointestinal microbiome, and their interactions may potentially explain the relationship between IS use, obesity and metabolic outcomes. While these elements are often studied in isolation, the potential interactions remain unexplored. Here, the current evidence of the relationship between IS use, obesity and metabolic outcomes is presented, and the potential roles for interactions with taste receptors and the gastrointestinal microbiota in modulating these relationships are explored.

## 1. Introduction

Intense sweeteners (IS) are many times sweeter than sugar, and therefore can be used in drastically smaller amounts, resulting in little to no energy contribution [1]. Sweeteners can be classified in multiple ways. IS are defined here to refer to artificial and natural sweeteners that are more than 30 times sweeter than sucrose [1]. IS are promoted as a healthful alternative to sugars and regarded as a safe means to combat the increasing incidence of obesity and diabetes when used within their acceptable daily intake (ADI) levels [1,2]. IS are commonly used; for example, in the United States, 41% of adults and 25% of children report daily IS consumption (*n* = 16,942) [3]. However, despite their reduced energy values, epidemiological and interventional evidence now suggests that regular IS consumption is linked to obesity and related issues [4,5,6,7,8].

IS have higher relative sweetness compared to sucrose (Table 1). This means much smaller amounts are required. This is due to higher binding affinity to sweet receptors. This higher intensity, combined with incomplete metabolism, accounts for their negligible energy contribution [9]. While there are over 115 plant compounds with reported sweetness [10], there are a limited number of commonly used natural and artificial sweeteners. The properties, relative sweetness and ADIs of IS currently approved for use in Australia and New Zealand [1], the United States [11] and Europe [12] are described in Table 1. Sugar alcohols are another important commercial sweetener; however, they are generally less sweet than sugar and often used for their other properties (thickening, stabilizing); as such, they are not included in this review [1].

IS, like sugars, bind to sweet taste receptors (T1Rs) on the tongue. Importantly, IS may also activate bitter oral receptors (T2Rs). Both T1Rs and T2Rs are G-protein coupled taste receptors. The T1R family consists of three different receptors. T1R1 and T1R3 heterodimerise detect umami, and T1R2 and T1R3 are involved in the detection of sweet compounds. The sweet receptor can detect different sweet ligands via different binding domains [13]. These receptors detect foods that are high energy and/or enjoyable [14]. Bitter compounds are detected by the T2R family of receptors. There are at least 25 functional human receptors capable of detecting hundreds of different bitter compounds [15]. Bitter receptors are involved in protection from the ingestion of bacteria and potential toxins, which relates to their relatively large numbers.

These receptors are also expressed throughout the gastrointestinal (GI) tract, where they are involved in the modulation of multiple metabolic processes, including glucose homeostasis, satiation and gut motility [16,17,18]. This has sparked recent interest in the roles for taste receptors in the development of obesity and related metabolic disorders [18,19,20]. Furthermore, the composition of the gut microbiome is known to be associated with the progression of obesity, diabetes and associated metabolic conditions [21,22,23,24]. Importantly, there is evidence to suggest that health issues like cardiovascular disease, metabolic syndrome and non-alcoholic fatty liver disease may also be associated with IS use [8,25], potentially via the activation of gastrointestinal taste receptors and subsequent altered hormone secretion, and/or perturbations to the intestinal microflora.

It is likely that there are multiple factors influencing the relationship between IS consumption, obesity and metabolic outcomes. However, these are yet to be fully elucidated. Importantly, reverse causality bias may explain some of these associations between IS use and metabolic dysfunction. However, as identified in an extensive review of the subject, the consistently identified association, coupled with the fact that the majority of studies take into account key potential confounders, suggests that reverse causality does not fully explain the increased risk [26]. Therefore, other biologically plausible mechanisms, such as the potential interactions between IS, taste receptors and the gastrointestinal microbiota, need to be considered. While the role of each of these elements in obesity and metabolic regulation have each previously been considered, their interactions largely remain to be investigated. Therefore, the current evidence of the relationship between IS use, obesity and metabolic outcomes is presented here, and the potential roles for interactions with taste receptors and the gastrointestinal microbiota in modulating these relationships are explored.

## 2. Weight and Intense Sweetener Consumption in Humans

IS consumption has been linked to weight gain and obesity in humans [5]. The first study to identify the association between artificial sweetener use and weight gain was a 1986 prospective cohort study of 78,694 women aged 50–69 [27]. This study found that artificial sweetener use was associated with increased body weight over a 1-year period. This study grouped women into quintiles based on initial body weight. Of the women who gained weight, IS-users in each quintile gained significantly more than non-users (+4.79 pounds compared to +4.17 pounds (*p <* 0.01), respectively, for the lowest quintile, and +8.19 pounds compared to +6.71 pounds (*p <* 0.001), respectively, for the highest quintile) [27]. It is important to note that obese/overweight individuals might consume more IS because of the widely accepted health benefits and a desire to lose weight. Nutrition data from an Australian cross-sectional study found that, while only 12.6% of normal-weight (*n* = 2678) adults consume IS daily, 25.7% of overweight (*n* = 2196) adults reported daily consumption of IS [28].

The majority of studies on IS and weight gain have focused on artificially sweetened beverages. A longitudinal study of 3682 adults in the United States examining weight changes over a 7–8 year period [29] found that overall there were significantly greater increases in body mass index (BMI) in those who consumed artificially sweetened beverages equivalent to approximately 0.5 BMI units (kg/m^2^). The same study noted that, in individuals consuming over 21 artificially sweetened beverages per week, the average change in BMI was greater than those who did not consume artificially sweetened beverages by more than 0.7 BMI units [29].

Other large studies have shown increases in BMI associated with consumption of IS-containing beverages, including an Australian cohort study (4791 women; 3103 men) observed over a 13-year period [30]. This study found that IS consumption was significantly associated with a greater 13-year BMI increase than people who reported consuming IS less than once a month (women: 0.52 kg/m^2^ (95% CI 0.38, 0.67); men: 0.28 kg/m^2^ (95% CI 0.15, 0.43)). Additionally, a nine year prospective study in 3682 adults highlighted a significant difference in the BMI of IS users (+1.48 kg/m) compared to non-users (+1.01 kg/m; *p <* 0.0001) [31]. Furthermore, it was determined that consuming water in the place of diet soft drink resulted in significantly greater weight loss (−8.8kg for water vs −7.6kg for the diet soft drink consumers) in an intervention study of 62 overweight and obese people [32]. Altogether, these studies suggest a link between consuming IS in diet soft drinks and weight gain.

Conversely, an interventional study in men and women (*n* = 303) over 12 weeks found that diet soft drink consumers lost more weight (−5.95 kg) than water consumers (−4.09 kg) over the study period [33]. Furthermore, a study of 163 obese women found that aspartame users regained less weight (+5.4 kg) than non-users (+9.4 kg) 2 years after initial weight loss (−10 kg) [34]. Importantly, multiple randomised control trials have found no significant association between IS-containing beverage use and weight [35,36,37]. Overall, there is conflicting evidence regarding the use of sweeteners and their relationship to weight. These inconsistent results may be explained by the unique structures of sweeteners and their varying potential to elicit downstream metabolic effects [38]. However, it is difficult to assess this in studies that focus on diet soft drink consumption and weight, as the type and quantities of sweeteners vary greatly between brands, and between drinks within brands. Importantly, the potential mechanisms involved in the relationship between weight gain and sweeter use likely relate to altered metabolic hormone secretion in response to IS, and subsequent metabolic dysfunction.

## 3. The Metabolic Effects of Intense Sweeteners

IS were developed to combat obesity and insulin resistance. In contrast, their use is now associated with not only potential weight gain, but altered glucose homeostasis, decreased satiety signalling, increased food intake and, interestingly, an altered gut microbiome [39,40]. IS appear to have widespread physiological effects, which have been extensively reviewed by Burke and Small, 2015 [41], Hunter et al., 2017 [38], Liauchonak et al., 2019 [42] and Swithers, 2013 [4]. In regard to metabolism, the weight gain and disrupted glucose homeostasis associated with IS consumption may be explained by the alteration of levels of glucagon-like peptide-1 (GLP-1). GLP-1 is a hormone that is secreted from intestinal epithelial endocrine cells in response to food intake. Its most notable role is in stimulating insulin secretion, but it is also involved in regulating appetite and food intake [43].

Altered GLP-1 secretion has been shown in rats (*n* = 72) exposed to saccharin [44]. These animals were on a high-fat, high-sugar diet, and provided with yoghurt sweetened with either saccharin (0.3% w/w) or glucose (20% w/w). The IS-consuming rats gained significantly more weight and had a significantly higher food intake (*p <* 0.05) after 28 days than the rats who consumed the glucose sweetened yoghurt, despite there being no significant differences between their starting weights. After these 28 days, fasting animals were given a glucose tolerance test (5 g of 20% glucose solution). At 6 and 18 min after glucose presentation, rats previously exposed to saccharin has significantly higher blood glucose (*p <* 0.05) and significantly lower GLP-1 (*p <* 0.05) than rats previously exposed to glucose sweetened yoghurt [44]. This study demonstrates the ability of IS exposure to affect weight, food intake, blood glucose and GLP-1 secretion in an animal model.

Disrupted glucose homeostasis was also identified in rodents in response to IS consumption. This study examined the effects of ad lib water compared to water with 5–7 mg/kg/d aspartame on rats (*n* = 44). These animals were also separated into standard chow diets and high-fat diets. Regardless of diet and body composition, aspartame-consuming animals had significantly elevated fasting blood glucose levels (*p <* 0.05) and impaired insulin function (*p <* 0.05) [45]. Further, in mice (*n* = 20) whose drinking water contained either water only, caloric sweeteners (10% glucose or 10% sucrose) or commercial artificial sweeteners (5% saccharin plus 95% glucose, 5% sucralose or 4% aspartame) made to 10% solutions, all IS-consuming groups developed significant glucose intolerance (*p <* 0.001) at 11 weeks [46]. Overall, these data suggest that IS consumption alters glucose homeostasis in rodents.

It is therefore not unexpected that IS consumption may be associated with the development of type 2 diabetes in humans. A French longitudinal study in 66,118 women identified that, over 14 years, 1369 of them developed type 2 diabetes. Importantly, there was a 68% increase in the risk of developing diabetes in that 14 years in women who consumed more than 603 mL per week of IS-containing beverages [47]. This study suggests that regular IS consumption may increase the risk of type 2 diabetes.

GLP-1 secretion has been shown to be altered in humans in response to IS consumption. This was demonstrated in a randomised study of 22 healthy volunteers which compared the ingestion of either 240 mL water or diet soda 10 min prior to a glucose load (75 g; 180 min) [48]. GLP-1 area under curve (AUC) was significantly higher in participants that consumed the diet soda AUC 24.0 +/− 15.2 pmol/L per 180 min) compared to water consumers (AUC 16.2 +/− 9.0 pmol/L per 180 min; *p =* 0.003). A similar effect was shown in another single-blinded, randomised human study. In this study, healthy participants (*n* = 16) were given either 24 mg sucralose in water (200 mL) or 200 mL water alone. The AUC of GLP-1 during a 75 g, 120 min glucose tolerance test was significantly higher following sucralose consumption (3192 ± 1108) compared to water (2463 ± 772; *p =* 0.04) [49]. Overall, these data suggest that IS consumption in the place of water may lead to altered GLP-1 secretion.

It has been further established that sucralose consumption increases plasma glucose levels and leads to altered insulin levels [50]. This was demonstrated in a randomised crossover design, where obese, insulin sensitive participants who did not use IS (*n* = 17) consumed either water (60 mL) or sucralose in water (48 mg; 60 mL) 10 min prior to a glucose load. Sucralose consumption was associated with increased insulin AUC (20 ± 8%; *p <* 0.03), increased peak plasma glucose levels (4.2 ± 0.2 vs. 4.8 ± 0.3 mmol/L; *p =* 0.03), increased peak insulin secretion rate (22 ± 7%; *p <* 0.02) and decreased insulin clearance (7 ± 4%; *p =* 0.04) [50]. Altogether, sucralose consumption significantly altered insulin responses in this cohort.

Appetite and food intake may also be affected by IS consumption. A study of 10 healthy subjects which compared the effects of four sweet preloads (40 g glucose, 40 g tagatose/isomalt mixture, 40 g 3-O-methylglucose or 60 mg sucralose) found that consuming sucralose before a meal resulted in reduced satiety [51]. In a similar study in 12 healthy adults, acesulfame K, aspartame and saccharin (240 mg, 162 mg and 145 mg, respectively) preloads were assessed for effects on food intake and appetite compared to water and glucose (50 g). It was found that the acesulfame K preload resulted in significantly higher food intake compared to the glucose preload (*p <* 0.05) [52]. Additionally, 30–60 min after the aspartame preload, there was significantly increased hunger and desire to eat compared to water (*p <* 0.05). These studies suggest that IS consumption before a meal may affect the release of satiety hormones, thereby altering food intake.

Overall, there is an established counterintuitive relationship between IS consumption and metabolic dysfunction. Differences in the metabolic effects of different sweeteners are likely to relate to their extensive structural differences [38,53] and subsequent ability to interact with gastrointestinal receptors. Interestingly, taste receptor genetics and altered receptor expression levels may be associated with obesity and related metabolic conditions [20]. This appears to relate to food preferences and intake. However, we propose that IS consumption may activate bitter and sweet receptors not only in the oral cavity but throughout the gastrointestinal tract, thereby modifying normal metabolic functions.

## 4. Oral Detection of Intense Sweeteners

In the oral cavity, taste receptors may be involved in determining dietary preferences and intake. It is important to note that taste perception is a complex and multifaceted trait. However, it is well-established that taste genotypes play a role in taste perception, and subsequent dietary intake. The most commonly studied human bitter taste gene is *TAS2R38*. The associated T2R38 receptor is responsible for the detection of phenylthiocarbamide (PTC) and 6-n-propyl-2-thiouracil (PROP) [54]. The *TAS2R38* genotype alone does not determine the ability to taste PTC and PROP [55]. However, it is still used as a general marker of taste acuity [56]. There are two common forms of the *TAS2R38* gene which arise from three single nucleotide polymorphisms (SNPs). These polymorphisms are part of a haplolock, and result in the amino acid substitutions proline-alanine-valine (PAV; associated with tasting PTC and prop) or alanine-valine-isoleucine (AVI; associated with not tasting PTC or PROP). This gives rise to three common *TAS2R* genotypes: PAV homozygotes (super tasters), heterozygotes (tasters) or AVI homozygotes (non-tasters). Findings from a recent systematic review of the genetic background of taste perception highlighted significant associations between three *TAS2R38* variants (rs713598, rs1726866, rs10246939) and bitter and sweet taste preferences [57]. Furthermore, multiple studies have identified a relationship between *TAS2R38* and BMI and/or food intake [58,59,60,61,62,63]. Importantly, some IS have the ability to activate both sweet (T1Rs) and bitter (T2Rs) taste receptors [64] (Table 2).

Oral taste receptor expression partly determines dietary intake, and expression may be modulated by dietary exposures or health conditions. A murine study which compared taste receptor expression between lean mice and mice with diet-induced obesity found that the sweet receptor (T1R2) and a bitter receptor (T2R118) were significantly down-regulated (*p <* 0.05) on the tongues of mice with diet induced obesity compared to wild type mice. Interestingly, the expression of these receptors in genetically obese mice was not significantly different from wild type mice [70]. This suggests that the expression of select taste receptors decreases in response to diet-induced obesity. Subsequently, this may lead to altered food intake (e.g., increased sugar consumption) and associated detrimental health effects. Therefore, it is hypothesised that a similar pattern of sweet receptor under-expression may also occur following IS consumption, resulting in the overconsumption of sugar.

Interestingly, IS consumers may have a higher sugar intake compared to water consumers. A cross sectional study on 7026 children in the US assessed differences in energy and macronutrient intake between consumers of IS-sweetened beverages, sugar-sweetened beverages or both, compared to water consumers. All three groups of sweetened beverage consumers reported significantly higher energy intake (*p <* 0.05), and a significantly higher intake of total sugars and added sugars (*p <* 0.05) [71]. Additionally, a study in 64 Canadian women, which grouped subjects based on regular sweetener intake (both IS and nutritive sweeteners), found that people who regularly consume larger amounts of sweeteners had a significantly greater preference for sweeter beverages (*p <* 0.05), which correlates with a higher intake of sugar [72]. There are several mechanisms by which consumption of sweeteners may increase the total intake of sugars [4,73], one of which may relate to altered taste receptor expression in response to IS consumption.

Genetic variation may also modulate dietary intake. Genotypes that lead to functional variations may result in reduced/improved taste sensation. In turn, this may lead to altered preferences [74]. In this way, *TAS1R* polymorphisms may alter the relative sweetness of IS [75,76], and therefore modify IS consumption between individuals [77,78]. Similarly, functional polymorphisms in *TAS2R* genes may also affect the bitterness of certain IS. In a study that genotyped participants (*n* = 108) and asked them to rate acesulphame K bitterness, it was shown that polymorphisms in the *TAS2R9* (rs3741845) and *TAS2R31* (rs10772423; rs10845293) genes were significantly associated with the perceived bitterness of acesulfame K (*p =* 0.009, *p =* 0.010 and *p =* 0.032, respectively) [65]. Further, this group modelled the combined effects of one *TAS2R9* genotype and one *TAS2R31* genotype, and found that this combination explained 13.4% of acesulphame K bitterness (*p <* 0.001) [65].

Similarly, in a study (*n* = 55) which analysed the relationship between *TAS2R43*-W35 and *TAS2R44*-W35 genotypes and saccharin bitterness, it was determined that individuals carrying at least one of these alleles were significantly more sensitive to the bitterness of saccharin than non-carriers (mean threshold 4.2 mM versus 25.7 mM, respectively; *p <* 0.0003) [79]. Furthermore, the combined hT2R43 and hT2R44 haplotype effect explains approximately 34% of the total variance in sensitivity to the bitterness of saccharin (*p <* 0.001) [79]. Overall, taste receptor expression and genotype appear to play a role in determining IS preferences and intake. These preferences may then influence diet and metabolic health status. Therefore, genetic variation in taste receptors may modulate the risk for obesity and associated conditions by determining IS intake.

## 5. The Role of Extra-Oral Receptors in Detecting and Responding to Intense Sweeteners

Taste receptors are not only expressed in the oral cavity but also throughout the body [80]. Recently, human taste receptors have been identified in vastly different organs, including the brain, heart, urethra, adipose and lungs [81]. Importantly, in the respiratory tract, T2Rs detect bacteria and bacterial quorum sensing molecules, and initiate protective immune responses [82,83,84,85]. Therefore, taste receptors within the gastrointestinal tract may also interact with the microbiota. In humans, there are at least 3 different T2Rs expressed in the gastrointestinal tissues [86]. Upon activation, extra-oral T2Rs regulate the secretion of metabolic hormones involved in appetite, energy intake, gut motility and glucose homeostasis [16,17,18,20,86,87,88,89,90,91]. These hormones include ghrelin (a hunger-inducing hormone), GLP-1, GIP (glucose-dependent insulinotropic polypeptide; involved in insulin regulation), CCK (cholecystokinin; involved in satiation and gut motility) and PYY (peptide tyrosine tyrosine; involved in gastric motility and food intake). These four hormones are secreted in different quantities along the gastrointestinal tract and work together to regulate food intake, glucose homeostasis and gut motility [92].

As well as the previously discussed effects of oral taste receptor stimulation and subsequently altered food intake, extra-oral T2R stimulation by IS may also have metabolic consequences. A study on human intestinal enteroendocrine cells demonstrated that not only is T2R9 is expressed in these cells but also a T2R9 ligand was found to stimulate GLP-1 secretion [87]. This study also looked at the relationship between the *TAS2R9* genotype and diabetes in 953 participants from the Amish Family Diabetes study. It was determined that the *TAS2R9*-rs3741845 T allele was significantly associated with a higher insulin AUC (858.2 ± 44.2 vs. 739.2 ±1 9.4 mmol/L; *p =* 0.006) and significantly higher glucose AUC (21.0 ± 0.3 vs. 19.8 ± 0.2 mmol/L; *p* = 0.036) [87]. This effect was likely due to altered GLP-1 secretion. Importantly, T2R9 is known to be activated by the IS acesulphame K (Table 1) [65]. Given this, we propose that IS like acesulphame K may disrupt glucose homeostasis by activating extra-oral bitter receptors and subsequently altering GLP-1 secretion.

When by-passing the oral cavity, IS elicit very different hormonal responses to caloric sweeteners. A randomised, double-blind crossover study in 12 healthy volunteers investigated the effects of intragastric administration of acesulfame K (220 mg) compared to nutritive sweeteners (50 g glucose and 25 g fructose) and water. It was determined that there was an initially stronger increase in satiety, followed by a significantly larger increase in hunger in response to acesulfame K treatment (*p* < 0.05) compared to water [93]. Furthermore, acesulfame K did not increase CCK secretions while the caloric sweeteners did. Overall, this study identified that gastrointestinal hormone secretion, and the subsequent inhibition of antral gastric motility and satiety in response to caloric sweeteners, did not occur in response to acesulfame K [93]. This suggests that IS may activate gastrointestinal T2Rs that are not activated by caloric sweeteners, which results in significantly different hormonal effects.

Sweet receptors may also be involved in the extra-oral detection of sweet compounds and release of GLP-1 [89]. Importantly, lactisole, a human sweet taste receptor antagonist, completely blocks the IS-mediated release of GLP-1 in vitro [89]. This supports a role for extra-oral sweet receptors in glucose homeostasis. Furthermore, a study in 72 mice that looked at the effect of sweeteners (sucrose 41.66 mg/mL, sucralose 4.16 mg/mL and stevia 4.16 mg/mL) on glycaemia and appetite found that, compared to water, sucralose treatment significantly reduced GIP secretion, glycaemia and food intake, but increased body weight. Further, stevia treatment increased the secretion of GIP, insulin, leptin, body weight and glycaemia [94]. These effects may be related to the potential activation of extra-oral T1Rs and the subsequent hormone release.

In humans, IS may also activate extra-oral T1Rs and subsequently alter intestinal hormone secretion. The role of gastrointestinal sweet taste receptors in the modulation of appetite has previously been identified in 35 healthy humans by analyzing the effects of lactisole (a sweet receptor blocker) on gut hormone secretion [95]. Following either intragastric administration of 75 g of glucose in 300 mL of water or 500 mL of a mixed liquid meal with or without lactisole or an intraduodenal perfusion of 29.3 g glucose/100 mL; rate: 2.5 mL/min for 180 min) or a mixed liquid meal (same rate) with or without lactisole, it was found that the lactisole treatments both resulted in significantly reduced GLP-1 and PPY secretion (*p* ≤ 0.05). Therefore, while artificial sweeteners like sucralose and aspartame and its derivatives are not known to stimulate bitter receptors, they may still alter the metabolism via the activation of extra-oral T1Rs.

In pancreatic tissues, sweet receptors detect IS and stimulate insulin secretion [96,97]. A study that looked at the effects of IS treatment on a mouse beta-cell line and mouse islets determined that T1R2 and T1R3 activation by sucralose, succharin and acesulfame K stimulated insulin secretion [96]. Furthermore, a murine study (*n* = 10) on diet-induced obesity found that a four percent IS (erythritol and aspartame) supplementation in drinking water resulted in significantly increased body adiposity and hyperinsulinemia compared to water controls (*p <* 0.05 for each) [98]. Together, these studies suggest that IS’ activation of pancreatic T1Rs may alter insulin secretion.

Both T1Rs and T2Rs are expressed in adipose tissue, and may detect and respond to IS in this tissue. Importantly, taste signaling molecules are involved in modulating leptin secretion [99]. It was identified in both human and mice precursor cells lines that treatments of between 2 mM and 4.5 mM saccharin and acesulfame K stimulated adipogenesis. Furthermore, in mature adipoctyes, IS treatment suppressed lipolysis [100]. Interestingly, this study noted that these effects were independent of T1Rs. However, both of these IS also stimulate T2Rs (Table 2), which was not explored in this study. Furthermore, in mice with diet-induced obesity, a 150 mg/kg/day dose of KDT501 (a T2R agonist) resulted in a significant reduction in the weight of adipose depots (*p <* 0.05) [19]. In humans, *TAS2R38* was overexpressed in adipocytes of obese (*n* = 32) compared to lean subjects (*n* = 18), and was found to be involved in the cell differentiation and delipidation processes [101]. Altogether, taste receptors appear to have roles in adipocyte metabolism that may be altered in response to IS.

Overall, in vitro and animal studies support the idea that ISs can stimulate hormone secretion from pancreatic, adipose and enteroendocrine cells. These studies have been extensively reviewed by others, including Brown and Rother, 2012 [102]; Bryant and Mclaughlin, 2016 [103]; Rother et al., 2018 [73]; and Han et al., 2019 [90]. However, it is important to note that there are limited studies confirming this in humans [87,93]. For example, while multiple studies report increased insulin concentrations after IS ingestion (sucralose or sucralose with acesulfame K) [50,104,105], others do not [51,106,107]. In two separate studies on the effects of diet soda consumption prior to a 75 g glucose load, it was found that IS consumption increases GLP-1 secretion. Furthermore, in nine type 1 diabetic subjects there was a 43% higher GLP-1 AUC for IS consumers compared to water (*p =* 0.02) [6], and in 22 healthy subjects, IS consumers had GLP-1 AUC of 24.0 +/− 15.2 pmol/L per 180 min versus carbonated water consumers (AUC 16.2 +/− 9.0 pmol/L per 180 min; *p =* 0.003) [48]. However, intragastric administration of sucralose (0.4 mM or 4 mM) did not affect GLP-1 or GIP release in seven healthy subjects compared to saline [106], and intraduodenal administration of sucralose (4 mM in 0.9% saline vs control 0.9% saline at 4 mL/min for 150 min) in 10 healthy people did not affect GLP-1 levels [108]. Additionally, a study of 12 healthy subjects demonstrated that intragastric infusions of either aspartame, acesulfame K or sucralose did not affect levels of GLP-1, PYY or ghrelin [109].

Overall, there is conflicting evidence from human trials regarding the effects of IS on the secretion of gastrointestinal hormones and the potential involvement of extra-oral taste receptors. The activation of gastrointestinal taste receptors may result in altered energy homeostasis and disrupted microbial function [5,20,102]. As these receptors have a key role in modulating metabolic functions, it is likely that IS-activation may lead to the subsequent secretion of key metabolic hormones. So far, this idea has been demonstrated in cell and mouse models, but not reliably replicated in human studies.

Differences between the type of IS are likely related to the vastly different IS structures. For example, while aspartame (294.3 g/mol) only binds to T1Rs [76], saccharin (183.19 g/mol) is known to activate T1Rs as well as at least three T2Rs [64,66]. Furthermore, given that taste receptor polymorphisms have been shown to affect taste sensitivity, potentially due to altered receptor binding affinity, we propose a similar occurrence in the gastrointestinal tract. Taste polymorphisms resulting in functional receptor changes may alter metabolic hormone secretion in the gastrointestinal tract.

## 6. Intense Sweeteners and The Gut Microbiome

One mechanism by which IS may alter metabolic function is via the modulation of bacterial composition. Conditions like obesity and insulin resistance are associated with certain gut microbial signatures and an overall decrease in bacterial diversity [21,22,23,110,111]. While not an extensive list, studies have shown that obese and overweight groups commonly show significant decreases in *Bifidobacterium* (*B. longum*; *B. adolescentis, B. animalis), Bacteroides (B. faecichinchillae*, *B. thetaiotaomicron and B. vulgatus*), *Lactobacillus* (*L. casei/paracasei and L. plantarum*), *Faecalibacterium prausnitzii* and *Akkermansia muciniphila*. Conversely, the microbial signatures of obese and overweight groups include increased Firmicutes (*Blautia hydrogenotorophica*, *Coprococcus catus*, *Eubacterium ventriosum*, *Ruminococcus bromii*, and *Ruminococcus obeum),* *Lactobacillus reuteri*, and potential pathogens like *Staphylococcus aureus, Escherichia Shigella* and *E. coli* and *Eubacterium rectale* [20,23,24,112,113]. In regard to diabetes, genera of *Bifidobacterium* (B*. bifidum, B. longum, B. infantis, B. animalis, B. pseudocatenulatum, B. breve*), *Bacteroides* (*B. intestinalis, B. 20–3 and B. vulgatus*), *Faecalibacterium prausnitzii, Akkermansia muciniphila* and *Roseburia* (*R. inulinivorans, Roseburia_272*,) were negatively associated with T2D. Additionally, *Ruminococcus gnavus* and *Fusobacterium nucleatum* were positively associated with T2D [21]. Overall, extensive studies have identified significant associations between certain metabolic conditions and gut microbial composition/function.

Importantly, IS use is strongly associated with perturbations to the intestinal microbiota in rodent models and humans [114]. It was recently identified in vitro that the artificial sweeteners saccharin, sucralose and acesulfame potassium have a direct bacteriostatic effect on common gut microflora (*E. coli* strains) [115]. This study also examined the effects of sucralose consumption (2.5% w/v in drinking water) on bacterial composition in mice. It was determined via 16S RNA sequencing that sucralose consumption induced dysbiosis in mice (significantly increased Firmicutes (*p* < 0.05) and significantly reduced Bacteroidetes (*p =* 0.117) compared to chow-only mice. Furthermore, this effect was exacerbated when sucralose was consumed in the context of a high-fat diet [115]. Overall, this study highlighted the detrimental effects of IS on commensal intestinal bacteria. In this way, IS consumption may exacerbate metabolic conditions. This idea has been demonstrated in a murine study which analysed the metabolic effects of low-dose aspartame treatment in rats. This study identified alterations to gut bacteria, along with elevated fasting glucose levels and insulin tolerance following an eight-week aspartame treatment (5–7 mg/kg/day, equivalent to two cans of diet soft drink; upper daily-recommended intake = 40–50 mg/kg/day) [45]. However, the mechanism by which this occurred remained unclear.

Similarly, there is also evidence to suggest that the natural intense sweetener stevia may disrupt the composition and function of the gut microbiome [116,117,118]. Stevia extracts are not metabolised in the upper gastrointestinal tract and therefore interact directly with colonic microbiota. It was shown in vitro that stevia extracts enhanced the growth of *bifidobacteria* and *lactobacilli* [119], and impedes the growth of *E. coli* strains in mice [115]. Furthermore, a recent murine study investigated the effects of maternal IS consumption on offspring. It was found that maternal stevia consumption (2–3 mg/kg/day) coupled with a high-fat diet (compared to high-fat diet only) leads to altered faecal microbiota in dams and offspring, and significantly increased offspring percent body fat at weaning (27.3 ± 1.3 vs. 21.4 ± 2 for female offspring and 24.7 ± 1.2 vs. 21.0 ± 1.2 for male offspring; *p <* 0.05 for both). Furthermore, upon receiving a microbial transplant from these offspring, germ-free mice had greater body fat and impaired glucose intolerance compared to obese wild type mice [116]. This study confirms that maternal IS consumption, paired with a high-fat diet, results in metabolic dysfunction in offspring who have not been themselves exposed to IS. Therefore, it is likely that the metabolic effects relate to the disrupted microbiome that is passed from dam to offspring.

A similar murine study also found that stevia consumption altered gut microbiota composition. In addition, this study demonstrated significantly increased caecal concentrations of the short-chain fatty acids acetate (*p =* 0.016) and valerate (*p =* 0.019). Interestingly, both acetate and valerate levels were significantly associated with increased fat mass and weight in response to stevia consumption (*p <* 0.05 for each) [117]. Overall, stevia may have detrimental effects on colonic microbial function, which may have consequences for metabolic derangements.

Furthermore, in a murine study that investigated the effects of IS consumption on body weight and food intake, it was shown that maximum artificial sweetener consumption (saccharin (10 mg/day, cyclamate 22 mg/day) resulted in significantly higher body weights compared to the control (*p <* 0.03 and *p <* 0.003, respectively). Interestingly, this did not correlate with altered food intake [120]. We propose that, as identified by Suez et al. [46], this weight increase may relate to alterations to the gut microbiota. Alternatively, the IS in this study may activate intestinal taste receptors, resulting in metabolic derangements.

**Figure 1:**
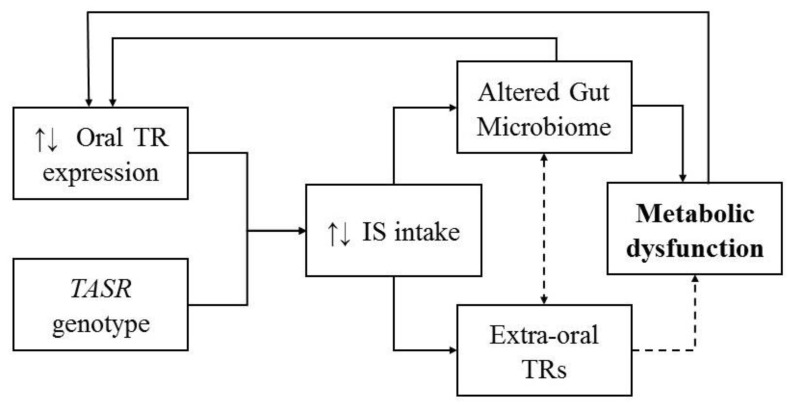
Potential interactions between taste receptors (TR), intense sweeteners (IS), the gut microbiome and metabolic conditions. Oral taste receptor expression levels, along with *TASR* genotypes, determine the palatability or aversiveness of sweeteners, which affects IS intake levels. IS may then activate gastrointestinal T1Rs and T2Rs, which may lead to altered metabolic hormone secretion. Some IS also have an effect on the composition and function of the intestinal microbiome, which may also lead to metabolic alterations. Interestingly, both an altered gut microbiome and certain metabolic disturbances may alter oral taste receptor expression levels. Finally, it is hypothesised that extra-oral taste receptor expression may be altered in response to intestinal dysbiosis, and this may impact the expression of extra-oral taste receptors, resulting in metabolic alterations.

In mice whose drinking water contained either water only, caloric sweeteners (10% glucose or 10% sucrose) or commercial artificial sweeteners (5% saccharin plus 95% glucose, 5% sucralose or 4% aspartame) made to 10% solutions, all IS-consuming groups developed significant glucose intolerance (*p <* 0.001) at 11 weeks [46]. Importantly, this effect was fully transferrable to germ-free mice and was shown to be eliminated by antibiotic treatment. This study also showed a similar effect of IS-induced dysbiosis on glucose intolerance in humans [46]. Data from 381 non-diabetic individuals was assessed for correlations between IS consumption and markers of metabolic syndrome. IS consumption was positively correlated with significantly higher levels of glycosylated haemoglobin (HbA1C%; *p <* 0.002). Furthermore, 172 randomly selected subjects from this cohort were selected for 16S sequencing. Significant positive correlations between IS consumption and multiple taxonomic classes were identified, including the Enterobacteriaceae family (*p <* 10^−6^), the Deltaproteobacteria class (*p <* 10^−5^) and the Actinobacteria phylum (*p <* 0.0003). In order to identify causation, seven volunteers who do not normally consume IS consumed 5 mg of commercial saccharin per kg (body weight) daily for a week. It was found that four out of seven volunteers (‘responders’) developed significantly impaired glycaemic responses (*p <* 0.001). Finally, stool samples from two of the responders were taken before day 1 and after day 7. When transferred to germ-free mice, stool from NAS responders induced significant glucose intolerance in recipient germ-free mice, compared to mice who received stool from before day 1 [46]. Overall, this study eloquently highlights the way in which IS consumption can alter glucose intolerance via modification of the intestinal microbiome (Figure 1).

The gut microbiome may be involved in the modulation of taste preferences and consumption of IS via manipulation of taste receptor expression [121]. Interestingly, taste receptor expression is altered following bariatric surgery. Along with satiety and food preferences, this surgery also alters the gastrointestinal microbiota, as reviewed by Miras and le Roux, 2013 [122]. This suggests a link between intestinal dysbiosis and altered taste preferences. Furthermore, germ-free mice have been shown to have a greater number of gastrointestinal sweet receptors, and had higher preferences for sweet-tasting foods compared to control mice [123]. Overall, the gut microbiota may alter taste preferences by manipulating taste receptor expression, which may subsequently affect microbial composition and alter the risk of metabolic disorders.

Metabolic conditions like insulin resistance and obesity, which are related to IS consumption and gut dysbiosis [5,21,22,23,46], are also associated with certain *TASR* genotypes [124] and altered taste receptor expression levels [31]. Importantly, taste receptors are activated by bacteria and bacterial compounds [20,80,81,82,85,125], and can also stimulate the secretion of anti-microbial agents [126]. Furthermore, receptor expression levels may change in response to altered bacterial compositions [20], resulting in altered metabolic functions. Given the crucial roles of taste receptors and the gut microbiome on metabolic health, we propose bidirectional interactions between these receptors and gastrointestinal microbes, which are altered in response to the consumption of IS. However, further investigations are needed to establish the mechanisms of this counter-intuitive relationship.

## 7. Conclusions

Taste receptors may represent a link between the use of IS, intestinal dysbiosis, weight gain and metabolic outcomes. While the metabolic effects of IS consumption on T1Rs have been explored in humans [102], the potential activation of bitter receptors and the downstream metabolic effects of that activation have been largely overlooked. Overall, IS may alter risk for metabolic disorders via interactions with taste receptors and intestinal microbiota. If this is the case, certain dietary or microbial interventions may be used for the prevention or treatment of metabolic conditions related to gastrointestinal dysbiosis and IS consumption. However, further studies are needed to confirm this association in humans and define the mechanisms.

## Figures and Tables

**Table 1:** Common intense sweeteners.

Name	Sweetener Type	Chemical Components [1]	Sweetness Relative to Sucrose [1]	AU ADI [1] (mg/ kg bw/d)	US ADI [11] (mg/ kg bw/ d)	EU ADI [12] (mg/ kg bw/ d)
Acesulphame K	Artificial	Acetoacetic acid and potassium	200×	15	15	9
Advantame	Artificial	Aspartame (below) and vanillin	20,000×	5	32.8	5
Alitame	Artificial	Aspartic acid and alanine	2000×	1	Not approved	Not approved
Aspartame	Artificial	Aspartic acid and phenylalanine	200×	40	50	40
Aspartame-acesulphame K salt	Artificial	Aspartame and acesulphame-K	350×	As respective elements	Not approved	As respective elements
Cyclamate	Artificial	Salt of cyclohexylsulfamic acid	30–50×	11	Not approved	7
Monk fruit extract	Natural	* Siraitia grosvenorii * fruit extract	250–400×	No ADI	No ADI	Not approved
Neotame	Artificial	Modified version of aspartame	7000–13,000×	2	0.3	2
Neohesperidine DC	Artificial	Modified Neohesperidin from citrus	1000×	Not approved	Not approved	5
Saccharin	Artificial	Forms: acid saccharin, sodium saccharin, potassium saccharin and calcium saccharin	300×	5	15	5
Stevia	Natural	Steviol glycosides from *Stevia rebaudiana*	200–300×	4	4	4
Sucralose	Artificial	Sucralose	600×	15	5	15
Thaumatin	Natural	*Thaumatococcus daniellii* fruit extract	2000–3000×	No ADI	Not approved	No ADI

AU, Australia; US, United States; EU, Europe; ADI, acceptable daily intake; bw, body weight; d, day.

**Table 2:** Common intense sweeteners that activate bitter taste receptors.

Name	Known to Activate T2Rs	Sources
Acesulphame potassium	T2R9, T2R43, T2R31	Allen et al. 2013 [65]; Kuhn et al., 2004 [64]; Meyerhof et al., 2010 [66]
Advantame	No	
Alitame	No	
Aspartame	No	
Aspartame-acesulphame salt	No	
Cyclamate	T2R1, T2R31, T2R38 T2R43	Behrens et al., 2017 [67]; Meyerhof et al., 2010 [66]
Monk fruit extract	No	
Neotame	No	
Neohesperidine DC	No	
Saccharin	T2R8, T2R43, T2R31	Kuhn et al., 2004 [64]; Meyerhof et al., 2010 [66]
Stevia	T2R4, T2R14	Acevedo et al., 2016 [68]; Hellfritsch et al., 2012 [69]
Sucralose	No	
Thaumatin	No	
		

## References

[1] Food Standards Australia and New Zealand Intense Sweeteners. http://www.foodstandards.gov.au/consumer/additives/Pages/Sweeteners.aspx.

[2] Franz M.J., Powers M.A., Leontos C., Holzmeister L.A., Kulkarni K., Monk A., Wedel N., Gradwell E. (2010). The Evidence for Medical Nutrition Therapy for Type 1 and Type 2 Diabetes in Adults. J. Am. Diet. Assoc..

[3] Sylvetsky A.C., Jin Y., Clark E.J., Welsh J.A., Rother K.I., Talegawkar S.A. (2017). Consumption of Low-Calorie Sweeteners among Children and Adults in the United States. J. Acad. Nutr. Diet..

[4] Swithers S.E. (2013). Artificial sweeteners produce the counterintuitive effect of inducing metabolic derangements. Trends Endocrinol. Metab..

[5] Pepino M.Y. (2015). Metabolic effects of non-nutritive sweeteners. Physiol. Behav..

[6] Brown R.J., Walter M., Rother K.I. (2012). Effects of Diet Soda on Gut Hormones in Youths With Diabetes. Diabetes Care.

[7] Hess E.L., Myers E.A., Swithers S.E., Hedrick V.E. (2018). Associations Between Nonnutritive Sweetener Intake and Metabolic Syndrome in Adults. J. Am. Coll. Nutr..

[8] Sylvetsky A.C., Rother K.I. (2018). Nonnutritive Sweeteners in Weight Management and Chronic Disease: A Review. Obesity.

[9] Magnuson B.A., Carakostas M.C., Moore N.H., Poulos S., Renwick A.G. (2016). Biological fate of low-calorie sweeteners. Nutr. Rev..

[10] ҪiçekS S. (2020). Structure-Dependent Activity of Plant-Derived Sweeteners. Molecules.

[11] Food and Drug Administration Additional Information about High-Intensity Sweeteners Permitted for Use in Food in the United States. https://www.fda.gov/food/food-additives-petitions/additional-information-about-high-intensity-sweeteners-permitted-use-food-united-states.

[12] European Commission Joint Research Centre S&S Table 7: Acceptable Daily Intake (ADI) of Sweeteners in the EU. https://ec.europa.eu/jrc/en/page/ss-table-7-acceptable-daily-intake-adi-sweeteners-eu-182968.

[13] Assadi-Porter F., Radek J., Rao H., Tonelli M. (2018). Multimodal Ligand Binding Studies of Human and Mouse G-Coupled Taste Receptors to Correlate Their Species-Specific Sweetness Tasting Properties. Molecules.

[14] Nelson G., Hoon M.A., Chandrashekar J., Zhang Y., Ryba N.J., Zuker C.S. (2001). Mammalian Sweet Taste Receptors. Cell.

[15] Go Y., Satta Y., Takenaka O., Takahata N. (2005). Lineage-Specific Loss of Function of Bitter Taste Receptor Genes in Humans and Nonhuman Primates. Genetics.

[16] Kochem M. (2017). Type 1 Taste Receptors in Taste and Metabolism. Ann. Nutr. Metab..

[17] Avau B., Depoortere I. (2015). The bitter truth about bitter taste receptors: Beyond sensing bitter in the oral cavity. Acta Physiol..

[18] Depoortere I. (2013). Taste receptors of the gut: Emerging roles in health and disease. Gut.

[19] Kok B., Galmozzi A., Littlejohn N., Albert V., Godio C., Kim W., Kim S., Bland J.S., Grayson N., Fang M. (2018). Intestinal bitter taste receptor activation alters hormone secretion and imparts metabolic benefits. Mol. Metab..

[20] Turner A., Veysey M., Keely S., Scarlett C.J., Lucock M., Beckett E.L. (2018). Interactions between Bitter Taste, Diet and Dysbiosis: Consequences for Appetite and Obesity. Nutrients.

[21] Gurung M., Li Z., You H., Rodrigues R., Jump D.B., Morgun A., Shulzhenko N. (2020). Role of gut microbiota in type 2 diabetes pathophysiology. EBioMedicine.

[22] Lin L., Zhang J. (2017). Role of intestinal microbiota and metabolites on gut homeostasis and human diseases. BMC Immunol..

[23] DeGruttola A.K., Low D., Mizoguchi A., Mizoguchi E. (2016). Current Understanding of Dysbiosis in Disease in Human and Animal Models. Inflamm. Bowel Dis..

[24] Gao R., Zhu C., Li H., Yin M., Pan C., Huang L., Kong C., Wang X., Zhang Y., Qu S. (2017). Dysbiosis Signatures of Gut Microbiota Along the Sequence from Healthy, Young Patients to Those with Overweight and Obesity. Obesity.

[25] Azad M.B., Abou-Setta A.M., Chauhan B.F., Rabbani R., Lys J., Copstein L., Mann A., Jeyaraman M.M., Reid A.E., Fiander M. (2017). Nonnutritive sweeteners and cardiometabolic health: A systematic review and meta-analysis of randomized controlled trials and prospective cohort studies. Can. Med. Assoc. J..

[26] Fowler S.P. (2016). Low-calorie sweetener use and energy balance: Results from experimental studies in animals, and large-scale prospective studies in humans. Physiol. Behav..

[27] Stellman S.D., Garfinkel L. (1986). Artificial sweetener use and one-year weight change among women. Prev. Med..

[28] Grech A., Kam C.O., Gemming L., Rangan A. (2018). Diet-Quality and Socio-Demographic Factors Associated with Non-Nutritive Sweetener Use in the Australian Population. Nutrients.

[29] Fowler S.P., Williams K., Resendez R.G., Hunt K.J., Hazuda H.P., Stern M.P. (2008). Fueling the Obesity Epidemic? Artificially Sweetened Beverage Use and Long-term Weight Gain. Obesity.

[30] Gearon E., Peeters A., Ng W., Hodge A.M., Backholer K. (2018). Diet and physical activity as possible mediators of the association between educational attainment and body mass index gain among Australian adults. Int. J. Public Health.

[31] Fowler L.J., Williams K., Hazuda H.P. (2015). Diet soda intake is associated with long-term increases in waist circumference in a biethnic cohort of older adults: The San Antonio Longitudinal Study of Aging. J. Am. Geriatr. Soc..

[32] Madjd A., Taylor M.A., Delavari A., Malekzadeh R., Macdonald I.A., Farshchi H.R. (2015). Effects on weight loss in adults of replacing diet beverages with water during a hypoenergetic diet: A randomized, 24-wk clinical trial. Am. J. Clin. Nutr..

[33] Peters J., Wyatt H.R., Foster G.D., Pan Z., Wojtanowski A.C., Veur S.S.V., Herring S.J., Brill C., Hill J.O. (2014). The effects of water and non-nutritive sweetened beverages on weight loss during a 12-week weight loss treatment program. Obesity.

[34] Blackburn G.L., Kanders B.S., Lavin P.T., Keller S.D., Whatley J. (1997). The effect of aspartame as part of a multidisciplinary weight-control program on short- and long-term control of body weight. Am. J. Clin. Nutr..

[35] Tate D., Turner-McGrievy G.M., Lyons E.J., Stevens J., Erickson K., Polzien K., Diamond M., Wang X., Popkin B.M. (2012). Replacing caloric beverages with water or diet beverages for weight loss in adults: Main results of the Choose Healthy Options Consciously Everyday (CHOICE) randomized clinical trial. Am. J. Clin. Nutr..

[36] Maersk M., Belza A., Stødkilde-Jørgensen H., Ringgaard S., Chabanova E., Thomsen H., Pedersen S.B., Astrup A., Richelsen B. (2011). Sucrose-sweetened beverages increase fat storage in the liver, muscle, and visceral fat depot: A 6-mo randomized intervention study. Am. J. Clin. Nutr..

[37] Hsieh M.-H., Chan P., Sue Y.-M., Liu J.-C., Liang T.H., Huang T.-Y., Tomlinson B., Chow M.S.S., Kao P.-F., Chen Y.-J. (2003). Efficacy and tolerability of oral stevioside in patients with mild essential hypertension: A two-year, randomized, placebo-controlled study. Clin. Ther..

[38] Hunter S., Reister E., Cheon E., Mattes R.D. (2019). Low Calorie Sweeteners Differ in Their Physiological Effects in Humans. Nutrients.

[39] Pearlman M., Obert J., Casey L. (2017). The Association between Artificial Sweeteners and Obesity. Curr. Gastroenterol. Rep..

[40] Brown R.J., De Banate M.A., Rother K.I. (2010). Artificial sweeteners: A systematic review of metabolic effects in youth. Pediatr. Obes..

[41] Burke M., Small D.M. (2015). Physiological mechanisms by which non-nutritive sweeteners may impact body weight and metabolism. Physiol. Behav..

[42] Liauchonak I., Qorri B., Dawoud F., Riat Y., Szewczuk M.R. (2019). Non-Nutritive Sweeteners and Their Implications on the Development of Metabolic Syndrome. Nutrients.

[43] Holst J.J. (2007). The Physiology of Glucagon-like Peptide 1. Physiol. Rev..

[44] Swithers S.E., Laboy A.F., Clark K., Cooper S., Davidson T.L. (2012). Experience with the high-intensity sweetener saccharin impairs glucose homeostasis and GLP-1 release in rats. Behav. Brain Res..

[45] Palmnäs M.S.A., Cowan T.E., Bomhof M.R., Su J., Reimer R.A., Vogel H.J., Hittel D.S., Shearer J. (2014). Low-Dose Aspartame Consumption Differentially Affects Gut Microbiota-Host Metabolic Interactions in the Diet-Induced Obese Rat. PLoS ONE.

[46] Suez J., Korem T., Zeevi D., Zilberman-Schapira G., Thaiss C.A., Maza O., Israeli D., Zmora N., Gilad S., Weinberger A. (2014). Artificial sweeteners induce glucose intolerance by altering the gut microbiota. Nature.

[47] Fagherazzi G., Vilier A., Sartorelli D.S., Lajous M., Balkau B., Clavel-Chapelon F. (2013). Consumption of artificially and sugar-sweetened beverages and incident type 2 diabetes in the Etude Epidémiologique auprès des femmes de la Mutuelle Générale de l’Education Nationale–European Prospective Investigation into Cancer and Nutrition cohort. Am. J. Clin. Nutr..

[48] Brown R.J., Walter M., Rother K.I. (2009). Ingestion of Diet Soda Before a Glucose Load Augments Glucagon-Like Peptide-1 Secretion. Diabetes Care.

[49] Temizkan S., Deyneli O., Yasar M., Arpa M., Gunes M., Yazici D., Sirikçi Ö., Haklar G., Imeryuz N., Yavuz D.G. (2014). Sucralose enhances GLP-1 release and lowers blood glucose in the presence of carbohydrate in healthy subjects but not in patients with type 2 diabetes. Eur. J. Clin. Nutr..

[50] Pepino M.Y., Tiemann C.D., Patterson B.W., Wice B.M., Klein S. (2013). Sucralose Affects Glycemic and Hormonal Responses to an Oral Glucose Load. Diabetes Care.

[51] Wu T., Zhao B.R., Bound M.J., Checklin H.L., Bellon M., Little T., Young R., Jones K.L., Horowitz M., Rayner C.K. (2011). Effects of different sweet preloads on incretin hormone secretion, gastric emptying, and postprandial glycemia in healthy humans. Am. J. Clin. Nutr..

[52] Rogers P.J., Carlyle J.-A., Hill A.J., Blundell J.E. (1988). Uncoupling sweet taste and calories: Comparison of the effects of glucose and three intense sweeteners on hunger and food intake. Physiol. Behav..

[53] Sylvetsky A.C., Blau J.E., Rother K.I. (2016). Understanding the metabolic and health effects of low-calorie sweeteners: Methodological considerations and implications for future research. Rev. Endocr. Metab. Disord..

[54] Kim U.-K., Jorgenson E., Coon H., Leppert M., Risch N., Drayna D. (2003). Positional Cloning of the Human Quantitative Trait Locus Underlying Taste Sensitivity to Phenylthiocarbamide. Science.

[55] Hayes J.E., Bartoshuk L.M., Kidd J.R., Duffy V.B. (2008). Supertasting and PROP Bitterness Depends on More than the TAS2R38 Gene. Chem. Senses.

[56] Tepper B.J., White E.A., Koelliker Y., Lanzara C., D’Adamo P., Gasparini P., D’Adamo P. (2009). Genetic Variation in Taste Sensitivity to 6-n-Propylthiouracil and Its Relationship to Taste Perception and Food Selection. Ann. N. Y. Acad. Sci..

[57] Diószegi J., Llanaj E., Ádány R. (2019). Genetic Background of Taste Perception, Taste Preferences, and Its Nutritional Implications: A Systematic Review. Front. Genet..

[58] Choi J.-H. (2019). Variation in the TAS2R38 Bitterness Receptor Gene Was Associated with Food Consumption and Obesity Risk in Koreans. Nutrients.

[59] Tepper B.J., Koelliker Y., Zhao L., Ullrich N.V., Lanzara C., D’Adamo P., Ferrara A., Ulivi S., Esposito L., Gasparini P. (2008). Variation in the Bitter-taste Receptor GeneTAS2R38, and Adiposity in a Genetically Isolated Population in Southern Italy. Obesity.

[60] Pawellek I., Grote V., Rzehak P., Xhonneux A., Verduci E., Stolarczyk A., Closa-Monasterolo R., Reischl E., Koletzko B. (2016). Association of TAS2R38 variants with sweet food intake in children aged 1–6 years. Appetite.

[61] Mikołajczyk-Stecyna J., Malinowska A., Chmurzyńska A. (2017). TAS2R38 and CA6 genetic polymorphisms, frequency of bitter food intake, and blood biomarkers among elderly woman. Appetite.

[62] Lumeng J.C., Cardinal T.M., Sitto J.R., Kannan S. (2008). Ability to taste 6-n-propylthiouracil and BMI in low-income preschool-aged children. Obesity.

[63] Ortega F.J., Agüera Z., Sabater M., Moreno-Navarrete J.M., Alonso-Ledesma I., Xifra G., Botas P., Delgado E., Jiménez-Murcia S., Fernández-García J.C. (2016). Genetic variations of the bitter taste receptor TAS2R38 are associated with obesity and impact on single immune traits. Mol. Nutr. Food Res..

[64] Kuhn C., Bufe B., Winnig M., Hofmann T., Frank O., Behrens M., Lewtschenko T., Slack J.P., Ward C.D., Meyerhof W. (2004). Bitter Taste Receptors for Saccharin and Acesulfame K. J. Neurosci..

[65] Allen A.L., McGeary J.E., Knopik V.S., Hayes J.E. (2013). Bitterness of the Non-nutritive Sweetener Acesulfame Potassium Varies With Polymorphisms in TAS2R9 and TAS2R31. Chem. Senses.

[66] Meyerhof W., Batram C., Kuhn C., Brockhoff A., Chudoba E., Bufe B., Appendino G., Behrens M. (2009). The Molecular Receptive Ranges of Human TAS2R Bitter Taste Receptors. Chem. Senses.

[67] Behrens M., Blank K., Meyerhof W. (2017). Blends of Non-caloric Sweeteners Saccharin and Cyclamate Show Reduced Off-Taste due to TAS2R Bitter Receptor Inhibition. Cell Chem. Boil..

[68] Acevedo W., González-Nilo F., Agosin E. (2016). Docking and Molecular Dynamics of Steviol Glycoside–Human Bitter Receptor Interactions. J. Agric. Food Chem..

[69] Hellfritsch C., Brockhoff A., Stähler F., Meyerhof W., Hofmann T.F. (2012). Human Psychometric and Taste Receptor Responses to Steviol Glycosides. J. Agric. Food Chem..

[70] Chao D.H.M., Argmann C., Van Eijk M., Boot R., Ottenhoff R., Van Roomen C., Foppen E., Siljee J.E., Unmehopa U.A., Kalsbeek A. (2016). Impact of obesity on taste receptor expression in extra-oral tissues: Emphasis on hypothalamus and brainstem. Sci. Rep..

[71] Sylvetsky A.C., Figueroa J., Zimmerman T., Swithers S.E., Welsh J.A. (2019). Consumption of low-calorie sweetened beverages is associated with higher total energy and sugar intake among children, NHANES 2011-2016. Pediatr. Obes..

[72] Mahar A., Duizer L. (2007). The Effect of Frequency of Consumption of Artificial Sweeteners on Sweetness Liking by Women. J. Food Sci..

[73] Rother K.I., Conway E.M., Sylvetsky A.C. (2018). How Non-nutritive Sweeteners Influence Hormones and Health. Trends Endocrinol. Metab..

[74] Bachmanov A., Bosak N.P., Lin C., Matsumoto I., Ohmoto M., Reed D., Nelson T.M. (2014). Genetics of Taste Receptors. Curr. Pharm. Des..

[75] Fernstrom J.D., Munger S.D., Sclafani A., De Araujo I.E., Roberts A., Molinary S. (2012). Mechanisms for sweetness. J. Nutr..

[76] Li X., Bachmanov A., Maehashi K., Li W., Lim R., Brand J.G., Beauchamp G.K., Reed D., Thai C., Floriano W. (2011). Sweet taste receptor gene variation and aspartame taste in primates and other species. Chem. Senses.

[77] García-Bailo B., Toguri C., Eny K.M., El-Sohemy A. (2009). Genetic Variation in Taste and Its Influence on Food Selection. OMICS J. Integr. Boil..

[78] Chamoun E., Mutch D., Allen-Vercoe E., Buchholz A.C., Duncan A.M., Spriet L.L., Haines J., Ma W.L.D. (2017). On behalf of the Guelph Family Health Study A review of the associations between single nucleotide polymorphisms in taste receptors, eating behaviors, and health. Crit. Rev. Food Sci. Nutr..

[79] Pronin A.N., Xu H., Tang H., Zhang L., Li Q., Li X. (2007). Specific Alleles of Bitter Receptor Genes Influence Human Sensitivity to the Bitterness of Aloin and Saccharin. Curr. Boil..

[80] Carey R.M., Lee R.J., Cohen N.A., Woodworth B., Poetker D., Reh D. (2016). Taste Receptors in Upper Airway Immunity. Adv. Oto Rhino Laryngol..

[81] Freund J., Lee R.J. (2018). Taste receptors in the upper airway. World J. Otorhinolaryngol. Head Neck Surg..

[82] Shah A.S., Ben-Shahar Y., Moninger T.O., Kline J.N., Welsh M.J. (2009). Motile Cilia of Human Airway Epithelia Are Chemosensory. Science.

[83] Tizzano M., Gulbransen B., Vandenbeuch A., Clapp T.R., Herman J.P., Sibhatu H.M., Churchill M., Silver W.L., Kinnamon S.C., Finger T. (2010). Nasal chemosensory cells use bitter taste signaling to detect irritants and bacterial signals. Proc. Natl. Acad. Sci. USA.

[84] Adappa N.D., Zhang Z., Palmer J.N., Kennedy D.W., Doghramji L., Lysenko A., Reed D., Scott T., Zhao N.W., Owens D. (2013). The bitter taste receptor T2R38 is an independent risk factor for chronic rhinosinusitis requiring sinus surgery. Int. Forum Allergy Rhinol..

[85] Lee R.J., Xiong G., Kofonow J.M., Chen B., Lysenko A., Jiang P., Abraham V., Doghramji L., Adappa N.D., Palmer J.N. (2012). T2R38 taste receptor polymorphisms underlie susceptibility to upper respiratory infection. J. Clin. Investig..

[86] Xie C., Wang X., Young R., Horowitz M., Rayner C.K., Wu T. (2018). Role of Intestinal Bitter Sensing in Enteroendocrine Hormone Secretion and Metabolic Control. Front. Endocrinol..

[87] Dotson C.D., Zhang L., Xu H., Shin Y.-K., Vigues S., Ott S.H., Elson A., Choi H.J., Shaw H., Egan J.M. (2008). Bitter Taste Receptors Influence Glucose Homeostasis. PLoS ONE.

[88] Janssen S., Laermans J., Verhulst P.-J., Thijs T., Tack J., Depoortere I. (2011). Bitter taste receptors and α-gustducin regulate the secretion of ghrelin with functional effects on food intake and gastric emptying. Proc. Natl. Acad. Sci. USA.

[89] Jang H.-J., Kokrashvili Z., Theodorakis M.J., Carlson O.D., Kim B.-J., Zhou J., Kim H.H., Xu X., Chan S.L., Juhaszova M. (2007). Gut-expressed gustducin and taste receptors regulate secretion of glucagon-like peptide-1. Proc. Natl. Acad. Sci. USA.

[90] Han P., Bagenna B., Fu M. (2018). The sweet taste signalling pathways in the oral cavity and the gastrointestinal tract affect human appetite and food intake: A review. Int. J. Food Sci. Nutr..

[91] Andreozzi P., Sarnelli G., Pesce M., Zito F.P., Alessandro A.D., Verlezza V., Palumbo I., Turco F., Esposito K., Cuomo R. (2015). The Bitter Taste Receptor Agonist Quinine Reduces Calorie Intake and Increases the Postprandial Release of Cholecystokinin in Healthy Subjects. J. Neurogastroenterol. Motil..

[92] Steinert R.E., Feinle-Bisset C., Asarian L., Horowitz M., Beglinger C., Geary N. (2017). Ghrelin, CCK, GLP-1, and PYY(3–36): Secretory Controls and Physiological Roles in Eating and Glycemia in Health, Obesity, and After RYGB. Physiol. Rev..

[93] Meyer-Gerspach A.C., Biesiekierski J.R., Deloose E., Clevers E., Rotondo A., Rehfeld J.F., Depoortere I., Van Oudenhove L., Tack J. (2018). Effects of caloric and noncaloric sweeteners on antroduodenal motility, gastrointestinal hormone secretion and appetite-related sensations in healthy subjects. Am. J. Clin. Nutr..

[94] Rosales C., Martinez-Carrillo B.E., Reséndiz-Albor A.A., Ramírez-Duran N., Valdés-Ramos R., Mondragón-Velásquez T., Escoto-Herrera J.A. (2018). Chronic Consumption of Sweeteners and Its Effect on Glycaemia, Cytokines, Hormones, and Lymphocytes of GALT in CD1 Mice. BioMed. Res. Int..

[95] Gerspach A.C., Steinert R.E., Schönenberger L., Graber-Maier A., Beglinger C. (2011). The role of the gut sweet taste receptor in regulating GLP-1, PYY, and CCK release in humans. Am. J. Physiol. Metab..

[96] Nakagawa Y., Nagasawa M., Yamada S., Hara A., Mogami H., Nikolaev V.O., Lohse M.J., Shigemura N., Ninomiya Y., Kojima I. (2009). Sweet Taste Receptor Expressed in Pancreatic β-Cells Activates the Calcium and Cyclic AMP Signaling Systems and Stimulates Insulin Secretion. PLoS ONE.

[97] Sternini C., Anselmi L., Rozengurt E. (2008). Enteroendocrine cells: A site of ‘taste’ in gastrointestinal chemosensing. Curr. Opin. Endocrinol. Diabetes Obes..

[98] Mitsutomi K., Masaki T., Shimasaki T., Gotoh K., Chiba S., Kakuma T., Shibata H. (2014). Effects of a nonnutritive sweetener on body adiposity and energy metabolism in mice with diet-induced obesity. Metabolism.

[99] Avau B., Bauters D., Steensels S., Vancleef L., Laermans J., Lesuisse J., Buyse J., Lijnen H.R., Tack J., Depoortere I. (2015). The Gustatory Signaling Pathway and Bitter Taste Receptors Affect the Development of Obesity and Adipocyte Metabolism in Mice. PLoS ONE.

[100] Simon B.R., Parlee S.D., Learman B.S., Mori H., Scheller E.L., Cawthorn W.P., Ning X., Gallagher K., Tyrberg B., Assadi-Porter F.M. (2013). Artificial Sweeteners Stimulate Adipogenesis and Suppress Lipolysis Independently of Sweet Taste Receptors. J. Boil. Chem..

[101] Cancello R., Micheletto G., Meta D., Lavagno R., Bevilacqua E., Panizzo V., Invitti C. (2020). Expanding the role of bitter taste receptor in extra oral tissues: TAS2R38 is expressed in human adipocytes. Adipocyte.

[102] Brown R.J., Rother K.I. (2012). Non-nutritive sweeteners and their role in the gastrointestinal tract. J. Clin. Endocrinol. Metab..

[103] Bryant C., McLaughlin J. (2016). Low calorie sweeteners: Evidence remains lacking for effects on human gut function. Physiol. Behav..

[104] Lertrit A., Srimachai S., Saetung S., Chanprasertyothin S., Chailurkit L.-O., Areevut C., Katekao P., Ongphiphadhanakul B., Sriphrapradang C. (2018). Effects of sucralose on insulin and glucagon-like peptide-1 secretion in healthy subjects: A randomized, double-blind, placebo-controlled trial. Nutrition.

[105] Sylvetsky A.C., Brown R.J., Blau J.E., Walter M., Rother K.I. (2016). Hormonal responses to non-nutritive sweeteners in water and diet soda. Nutr. Metab..

[106] Ma J., Bellon M., Wishart J.M., Young R., Blackshaw A., Jones K.L., Horowitz M., Rayner C.K. (2009). Effect of the artificial sweetener, sucralose, on gastric emptying and incretin hormone release in healthy subjects. Am. J. Physiol. Liver Physiol..

[107] Brown A.W., Brown M.M.B., Onken K.L., Beitz D.C. (2011). Short-term consumption of sucralose, a nonnutritive sweetener, is similar to water with regard to select markers of hunger signaling and short-term glucose homeostasis in women. Nutr. Res..

[108] Ma J., Chang J., Checklin H.L., Young R., Jones K.L., Horowitz M., Rayner C.K. (2010). Effect of the artificial sweetener, sucralose, on small intestinal glucose absorption in healthy human subjects. Br. J. Nutr..

[109] Steinert R.E., Frey F., Töpfer A., Drewe J., Beglinger C. (2011). Effects of carbohydrate sugars and artificial sweeteners on appetite and the secretion of gastrointestinal satiety peptides. Br. J. Nutr..

[110] Bäckhed F., Ding H., Wang T., Hooper L.V., Koh G.Y., Nagy A., Semenkovich C.F., Gordon J.I. (2004). The gut microbiota as an environmental factor that regulates fat storage. Proc. Natl. Acad. Sci. USA.

[111] Karlsson F., Tremaroli V., Nookaew I., Bergström G., Behre C.J., Fagerberg B., Nielsen J., Bäckhed F. (2013). Gut metagenome in European women with normal, impaired and diabetic glucose control. Nature.

[112] Gomes A.C., Hoffmann C., Mota J.F. (2018). The human gut microbiota: Metabolism and perspective in obesity. Gut Microbes.

[113] Castañer O., Goday A., Park Y.-M.M., Lee S.H., Magkos F., Shiow S.-A.T.E., Schröder H. (2018). The Gut Microbiome Profile in Obesity: A Systematic Review. Int. J. Endocrinol..

[114] Gültekin F., Oner M.E., Savaş H.B., Dogan B. (2019). Food additives and microbiota. North. Clin. Istanb..

[115] Wang Q.-P., Browman D., Herzog H., Neely G.G. (2018). Non-nutritive sweeteners possess a bacteriostatic effect and alter gut microbiota in mice. PLoS ONE.

[116] Nettleton J.E., Cho N.A., Klancic T., Nicolucci A.C., Shearer J., Borgland S.L., Johnston L.A., Ramay H.R., Tuplin E.N., Chleilat F. (2020). Maternal low-dose aspartame and stevia consumption with an obesogenic diet alters metabolism, gut microbiota and mesolimbic reward system in rat dams and their offspring. Gut.

[117] Nettleton J.E., Klancic T., Schick A., Choo A.C., Shearer J., Borgland S.L., Chleilat F., Mayengbam S., Reimer R.A. (2019). Low-Dose Stevia (Rebaudioside A) Consumption Perturbs Gut Microbiota and the Mesolimbic Dopamine Reward System. Nutrients.

[118] Ruiz-Ojeda F.J., Plaza-Díaz J., Sáez-Lara M.J., Gil A. (2019). Effects of Sweeteners on the Gut Microbiota: A Review of Experimental Studies and Clinical Trials. Adv. Nutr..

[119] Lopes S.M.S., Francisco M.G., Higashi B., Almeida R.R., Krausová G., Pilau E., Gonçalves J.E., Gonçalves R.A.C., De Oliveira A.J.B. (2016). Chemical characterization and prebiotic activity of fructo-oligosaccharides from Stevia rebaudiana (Bertoni) roots and in vitro adventitious root cultures. Carbohydr. Polym..

[120] Polyák É., Gombos K., Hajnal B., Bonyár-Müller K., Szabó S., Gubicskó-Kisbenedek A., Marton K., Ember I. (2010). Effects of artificial sweeteners on body weight, food and drink intake. Acta Physiol. Hung..

[121] Alcock J., Maley C.C., Aktipis C.A. (2014). Is eating behavior manipulated by the gastrointestinal microbiota? Evolutionary pressures and potential mechanisms. BioEssays.

[122] Miras A.D., Le Roux C.W. (2013). Mechanisms underlying weight loss after bariatric surgery. Nat. Rev. Gastroenterol. Hepatol..

[123] Swartz T.D., Duca F.A., De Wouters T., Sakar Y., Covasa M. (2011). Up-regulation of intestinal type 1 taste receptor 3 and sodium glucose luminal transporter-1 expression and increased sucrose intake in mice lacking gut microbiota. Br. J. Nutr..

[124] Beckett E.L., Martin C., Yates Z., Veysey M., Duesing K., Lucock M. (2014). Bitter taste genetics—The relationship to tasting, liking, consumption and health. Food Funct..

[125] Carey R.M., Workman A.D., Chen B., Adappa N.D., Palmer J.N., Kennedy D.W., Lee R.J., Cohen N.A. (2015). Staphylococcus aureus triggers nitric oxide production in human upper airway epithelium. Int. Forum Allergy Rhinol..

[126] Yan C.H., Hahn S., McMahon D., Bonislawski D., Kennedy D.W., Adappa N.D., Palmer J.N., Jiang P., Lee R.J., Cohen N.A. (2017). Nitric oxide production is stimulated by bitter taste receptors ubiquitously expressed in the sinonasal cavity. Am. J. Rhinol. Allergy.

